# Binding of *Staphylococcus aureus* Protein A to von Willebrand Factor Is Regulated by Mechanical Force

**DOI:** 10.1128/mBio.00555-19

**Published:** 2019-04-30

**Authors:** Felipe Viela, Valeria Prystopiuk, Audrey Leprince, Jacques Mahillon, Pietro Speziale, Giampiero Pietrocola, Yves F. Dufrêne

**Affiliations:** a Institute of Life Sciences, Université Catholique de Louvain, Louvain-la-Neuve, Belgium; b Laboratory of Food and Environmental Microbiology, Earth and Life Institute, Université Catholique de Louvain, Louvain-la-Neuve, Belgium; c Department of Molecular Medicine, Unit of Biochemistry, University of Pavia, Pavia, Italy; d Department of Industrial and Information Engineering, University of Pavia, Pavia, Italy; e Walloon Excellence in Life sciences and Biotechnology (WELBIO), Liege, Belgium; Centre International de Recherche en Infectiologie; Harvard Medical School

**Keywords:** adhesion, *Staphylococcus aureus*, atomic force microscopy, mechanical force, von Willebrand factor

## Abstract

Binding of Staphylococcus aureus to the large plasma glycoprotein von Willebrand factor (vWF) is controlled by hydrodynamic flow conditions. Currently, we know little about the molecular details of this shear-stress-dependent interaction. Using single-molecule atomic force microscopy, we demonstrate that vWF binds to the S. aureus surface protein A (SpA) via a previously undescribed force-sensitive mechanism. We identify an extremely strong SpA-vWF interaction, capable of withstanding forces of ∼2 nN, both in laboratory and in clinically relevant methicillin-resistant S. aureus (MRSA) strains. Strong bonds are activated by mechanical stress, consistent with flow experiments revealing that bacteria adhere in larger amounts to vWF surfaces when the shear rate is increased. We suggest that force-enhanced adhesion may involve conformational changes in vWF. Under force, elongation of vWF may lead to the exposure of a high-affinity cryptic SpA-binding site to which bacteria firmly attach. In addition, force-induced structural changes in the SpA domains may also promote strong, high-affinity binding. This force-regulated interaction might be of medical importance as it may play a role in bacterial adherence to platelets and to damaged blood vessels.

## Abstract (precis)

Staphylococcus aureus protein A (SpA) binds to von Willebrand factor (vWF) under flow. While vWF binding to SpA plays a role in S. aureus adherence to platelets and endothelial cells under shear stress, the molecular basis of this stress-dependent interaction has not yet been elucidated. Here we show that the SpA-vWF interaction is regulated by a new force-dependent mechanism. The results suggest that mechanical extension of vWF may lead to the exposure of a high-affinity cryptic SpA-binding site, consistent with the shear force-controlled functions of vWF. Moreover, strong binding may be promoted by force-induced structural changes in the SpA domains. This study highlights the role of mechanoregulation in controlling the adhesion of S. aureus and shows promise for the design of small inhibitors capable of blocking colonization under high shear stress.

## INTRODUCTION

Staphylococcus aureus is a leading cause of endovascular infections, such as infective endocarditis or heart valve prosthetic infection (1, 2). During endovascular infections, the pathogen needs to attach to the endothelium and to withstand the shear stress of flowing blood. S. aureus binds to endothelium under flow via von Willebrand factor (vWF) (3–5), a large multimeric glycoprotein produced by endothelial cell layers and stored in Weibel-Palade bodies and α-granules of platelets. The basic vWF monomer is a 2,050-amino-acid protein containing four types of repeat domains, A, B, C, and D, which are arranged in the sequence D1-D2-D′-D3-A1-A2-A3-D4-B1-B2-B3-C1-C2-CK in the mature protein. vWF is a multifunctional protein interacting with a variety of ligands. The D′D3 domain binds factor VIII, the A1 domain binds to platelet GPIb receptor, heparin, and possibly collagen, the A2 domain contains the buried cleavage site for the specific ADAM13 protease, the A3 domain binds collagen, and the C1 domain contains an RGD motif involved in binding platelet integrin αIIbβ3. After secretion, vWF is elongated as a result of blood flow and forms fibers on endothelial layers. In contrast to the globular state, vWF under shear exposes cryptic glycoprotein platelet binding sites to which platelets bind with high affinity. The transition from a compact into an extended conformation leads to the activation of the vWF A1 domain to bind platelets (6).

S. aureus interacts with vWF using the bacterial cell surface protein A (SpA) (7, 8), the secreted coagulase Willebrand factor-binding protein (vWFbp) (8, 9), and clumping factor A (ClfA) (5). SpA is a 55-kDa protein (which including the signal peptide, may differ between isolates) comprising five N-terminal tandemly linked triple-helical bundle domains, each of which binds to different ligands, including the IgG Fc region, the tumor necrosis factor receptor 1 (TNFR-1) (10), and the epidermal growth factor receptor (EGFR) (11), followed by the repeat containing the Xr and the nonrepetitive Xc regions. SpA also binds with high affinity to the vWF A1 domain and to a lesser extent to the D′/D3 domains (12).

There is increasing evidence that binding of vWF to S. aureus is controlled by hydrodynamic flow. Under fluid shear stress, ClfA binds via vWFbp to vWF present on activated endothelial cells (4, 5). In addition, ClfA binds in a shear-dependent manner to fibrinogen, which, in turn, interacts with integrins expressed by endothelial cells, suggesting a dual role of the adhesin in S. aureus adhesion to endothelium (13). Shear flow has also been shown to increase SpA-dependent bacterial attachment to endothelial cell monolayers and to the extracellular matrix (3, 8, 12). While these flow experiments show that S. aureus-vWF adhesion is modulated by shear stress, the underlying molecular mechanisms are poorly understood. Here we unravel the strength and dynamics of the SpA-vWF interaction using single-molecule atomic force microscopy (AFM) (14). We find that SpA mediates S. aureus adhesion to vWF via a novel force-dependent mechanism involving conformational changes in the vWF and SpA molecules. The stress-induced activation of the SpA-vWF interaction may be critical for bacterial adherence to platelets and to damaged blood vessels.

## RESULTS

### SpA favors bacterial adhesion to vWF, both *in vitro* and on endothelial cells.

**FIG 1:**
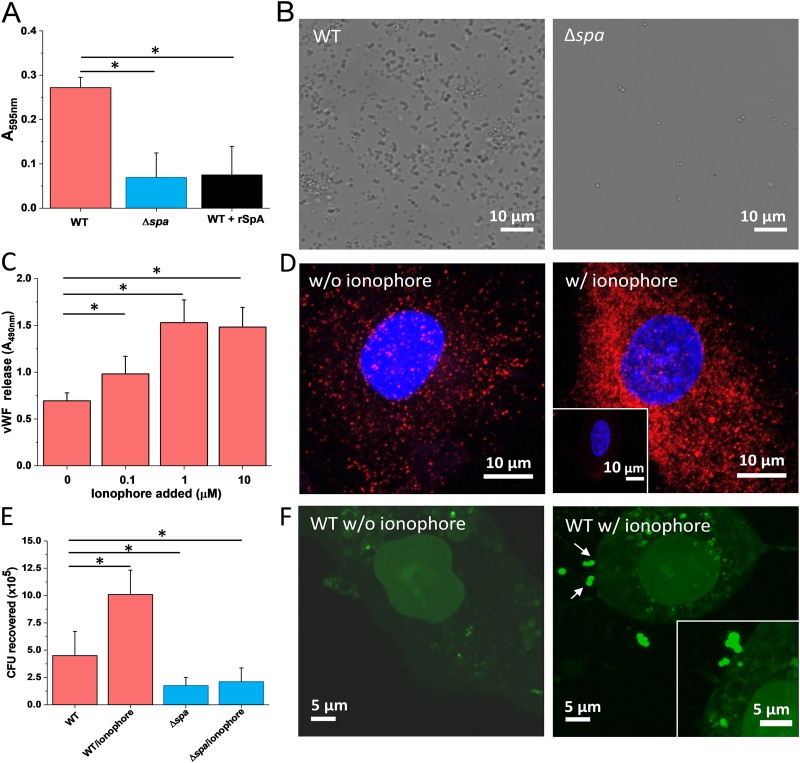
Attachment of S. aureus to immobilized vWF and to endothelial cells. (A) Microtiter wells coated with vWF were incubated with S. aureus Newman WT and Newman Δ*spa* bacteria, rinsed, and stained with crystal violet, and the absorbance at 595 nm was measured in an ELISA plate reader. Attachment of WT bacteria was also performed in the presence of 2 μg soluble SpA. (B) Optical microscopy images of S. aureus Newman WT and Newman Δ*spa* bacteria adhering to vWF-coated substrates. (C) vWF release by endothelial cells. Confluent HUVEC monolayers were incubated with increasing concentrations of calcium ionophore A23187 for 10 min. The vWF released in the extracellular matrix was determined with rabbit anti-vWF polyclonal antibody followed by HRP-conjugated goat anti-rabbit. (D) Imaging of vWF release. Shown are confocal microscopy images of HUVECs before (left) and after (right) treatment with calcium ionophore. Staining with mouse anti-vWF antibody and secondary goat anti-mouse Alexa Fluor 647 antibody documents the release of large amounts of vWF upon ionophore treatment (vWF is in red, and the nucleus is in blue). The inset shows a control experiment in which the primary antibody was missing. (E) Endothelial cell adhesion assays. Confluent HUVECs were incubated with S. aureus Newman WT and Newman Δ*spa* bacteria for 90 min, in the presence or absence of 1 μM ionophore. The number of adhering bacteria was determined as described in Materials and Methods. (F) Imaging of bacterial-endothelial cell adhesion. Confluent HUVECs, treated (right) or not (left) with ionophore, were incubated with S. aureus Newman WT bacteria stained with the BacLight viability kit, rinsed, and imaged by confocal microscopy. The arrows indicate bacteria adhering to the endothelial cell surface. For all plots, means and SD of results from two independent experiments, each performed in triplicate, are presented. Statistically significant difference is indicated (Student's *t* test; *, *P* < 0.05).

To confirm the role of SpA in promoting bacterial adhesion to vWF, we studied the attachment of the S. aureus Newman strain, known to largely express SpA (15–17), onto vWF immobilized on microtiter wells. Wild-type (WT) bacteria adhered to vWF in large amounts, whereas bacteria from the Newman Δ*spa* strain lacking the adhesin did to a much lower extent (Fig. 1A). Adhesion of WT bacteria was inhibited by soluble SpA, demonstrating the specificity of interaction (Fig. 1A). Optical microscopy imaging confirmed that SpA is involved in vWF-dependent adhesion since WT bacteria, unlike Δ*spa* ones, extensively colonized vWF-covered glass substrates (Fig. 1B). Note that this optical assay confirms proper expression of SpA, but should not be considered quantitative as some shear stress was applied during rinsing.

We also investigated bacterial adhesion to vWF in the extracellular matrix (ECM) of human umbilical vein endothelial cells (HUVECs). To promote vWF exposure, endothelial cells were allowed to spread on glass surfaces and then treated with the calcium ionophore A23187. Upon ionophore activation, the Weibel-Palade bodies are transported to the cell surface, releasing vWF, which is temporarily retained on the cell surface, thus making it available for interaction with S. aureus (18). Incubating monolayers with a specific vWF antibody, we found that the vWF release increased with the concentration of ionophore added (Fig. 1C). In line with this, confocal microscopy revealed that treatment with the ionophore largely increased the exposure of vWF in the ECM (Fig. 1D). Microscopic adhesion assays showed that WT bacteria attached in much larger amounts to HUVECs when these were activated with ionophore (Fig. 1E and F). The Δ*spa* strain showed much lower adhesion than the WT, both with and without ionophore. Some residual attachment was observed, suggesting that other adhesins may play a role. The above observations show that, under our conditions, S. aureus binds to vWF both *in vitro* and on endothelial cells primarily via SpA proteins. The low adhesion of the Δ*spa* strain is in contrast with the results of Claes et al. (4, 5) showing that under high fluid shear stress, ClfA binds via vWFbp to vWF on activated endothelial cells. However, our adhesion assays were not performed at high stress. It is also possible that under our conditions, bacteria did not secrete much vWFbp proteins as the cells were centrifuged before used. Additionally, the involvement of vWFbp could vary, depending on the secreted amount of vWFbp, its rebinding to ClfA, and the availability of surface-expressed ClfA to interact with this protein.

### Adhesion forces between single S. aureus bacteria and vWF.

**FIG 2:**
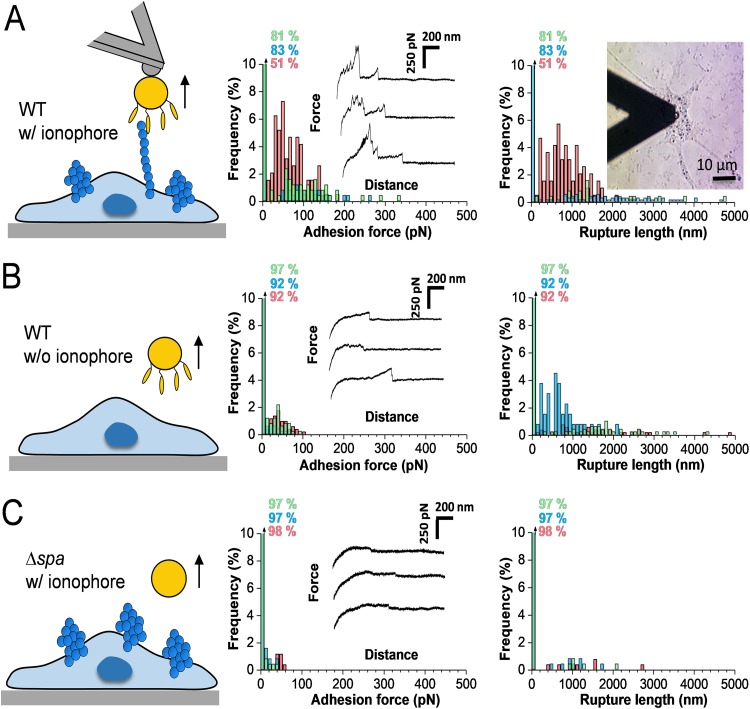
Adhesion forces between S. aureus and vWF in the extracellular matrix. (A) Maximum adhesion force (left) and rupture length (right) histograms with representative retraction force profiles (insets) obtained by recording force-distance curves in HEPES buffer between Newman bacteria and ionophore-treated endothelial cells. Data from a total of 1,536 curves from 3 different bacterial-HUVEC cell pairs are shown. (B) Force data collected between Newman bacteria and nontreated endothelial cells (1,536 curves from 3 cell pairs). (C) Force data collected between Newman Δ*spa* bacteria and ionophore-treated endothelial cells (1,536 curves from 3 cell pairs). The percentage shown represents the proportion of nonadhesive events. For data on more cells, see Table S1.

We measured the interaction forces between S. aureus and vWF in the ECM of HUVECs using AFM (Fig. 2). Shown in Fig. 2A are the adhesion forces, rupture lengths, and representative force curves obtained between single Newman bacteria and ionophore-treated endothelial cells (3 representative bacterial-HUVEC cell pairs [for more data, see Table S1 in the supplemental material]). Force curves (27% ± 18%) showed multiple adhesion peaks of a magnitude of 114 ± 55 pN (mean ± standard deviation [SD]; *n *= 684 adhesive curves from 10 cell pairs [Table S1]) and 1,679 ± 974-nm rupture lengths (*n *= 684). Much weaker adhesion was observed with native, untreated endothelial cells (Fig. 2B; Table S1), or when using the Newman Δ*spa* mutant (Fig. 2C; Table S1), demonstrating that the ∼100-pN forces are associated with specific SpA-vWF bonds. The shape of adhesive curves did not change over time, indicating that the bacterial cell probes were not denatured or contaminated during repeated force curve measurements. Force profiles did not show force plateaus, meaning membrane tethers were not formed upon pulling. This indicates that bacteria did not directly interact with the endothelial cell membrane, but rather with loosely anchored vWF. As the vWF-ECM interaction is probably the weak side of the three-component complex, under force, the vWF-ECM bond will rupture before the SpA-vWF bond.

10.1128/mBio.00555-19.4 TABLE S1 Adhesion forces between S. aureus and vWF in the ECM of endothelial cells. Probability of adhesion (*P*_adh_ = % of curves with adhesion forces), maximum adhesion force (*F*_adh_), and rupture length (*L*_rupt_) were measured for comparison between single Newman WT or Newman Δ*spa* bacteria and HUVECs treated or not with calcium ionophore. Download Table S1, DOCX file, 0.1 MB. Copyright © 2019 Viela et al. 2019 Viela et al. This content is distributed under the terms of the Creative Commons Attribution 4.0 International license.

**FIG 3:**
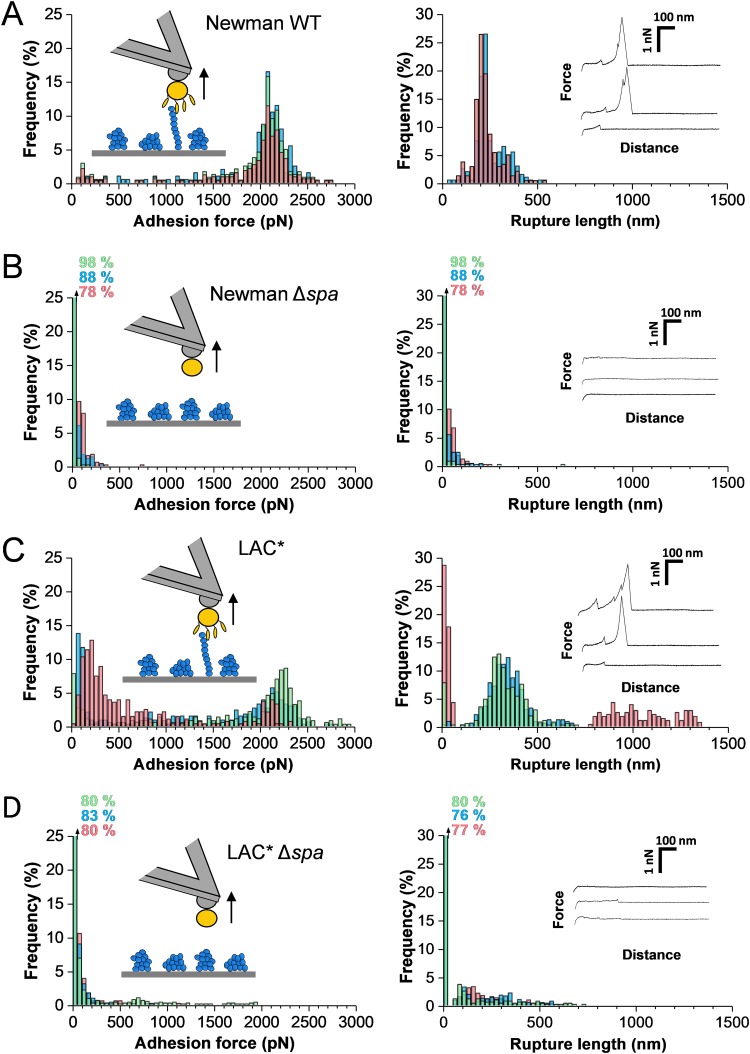
Adhesion forces between S. aureus and immobilized vWF. (A and B) Maximum adhesion force (left) and rupture length (right) histograms with representative retraction force profiles obtained by recording force distance curves in PBS between Newman WT (A) or Newman Δ*spa* (B) bacteria and vWF-coated substrates. (C and D) Force data obtained for the clinically relevant LAC* strain (C) and its LAC* Δ*spa* mutant (D). For each panel, data from three representative bacterial cells are shown.

**FIG 4:**
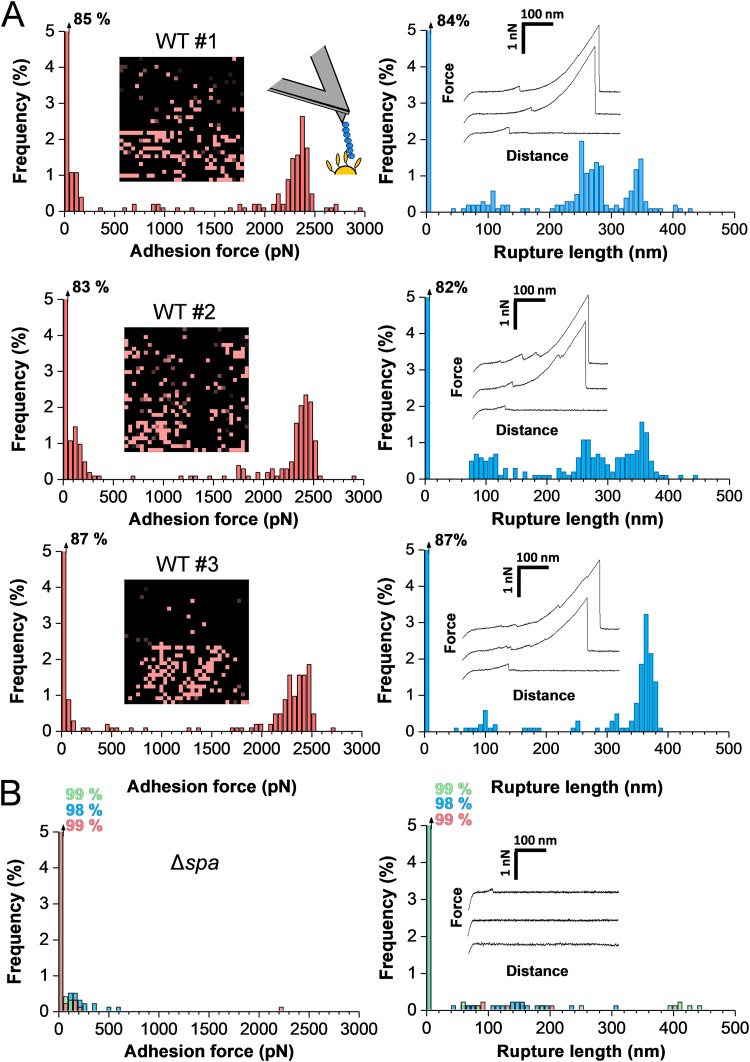
Strength of single SpA-vWF bonds. (A) Maximum adhesion force histograms (left) with force maps (insets; image size = 500 nm) and rupture length histograms (right) with representative retraction force profiles (insets) obtained by recording force-distance curves in PBS between 3 different Newman WT bacteria and vWF-modified AFM tips. (B) Data obtained under the same conditions for Newman Δ*spa* bacteria. (Data from 3 cells are merged.)

To unravel the strength of the SpA-vWF interaction, we therefore analyzed the forces between S. aureus and vWF immobilized on solid substrates. As shown in Fig. 3A, force profiles between single Newman bacteria and vWF featured strong adhesion peaks with mean forces of 2,116 ± 235 pN (mean ± SD; *n *= 659 adhesive curves from 3 cells [for more cells, see Fig. S1A in the supplemental material]) and mean rupture length of 213 ± 92 nm (*n *= 659). These forces are specific to the SpA-vWF interaction as they were lacking in Newman Δ*spa* cells (Fig. 3B). High forces reflect the rupture of single bonds rather than multiple weak bonds as such sharp force distributions were observed in multiple cells, as well as in single-molecule experiments with vWF-coated AFM tips (Fig. 4 [see below]). The bonds ruptured at ∼200 to 300 nm, which is in the range of the length of the unfolded SpA protein (∼180 nm, assuming the adhesin is made of ∼500 residues [19]). However, it is likely that vWF will also contribute to protein extensions (see below), meaning that SpA would not be completely unfolded.

10.1128/mBio.00555-19.1 FIG S1 Adhesion forces between S. aureus and immobilized vWF. (A and B) Maximum adhesion force (left) and rupture length (right) histograms obtained by recording force-distance curves in PBS between three additional Newman WT (A) or LAC* (B) bacteria and vWF-coated substrates. Download FIG S1, DOCX file, 1.6 MB. Copyright © 2019 Viela et al. 2019 Viela et al. This content is distributed under the terms of the Creative Commons Attribution 4.0 International license.

Are the interaction forces observed on the Newman laboratory strain clinically relevant? We found that cells from the methicillin-resistant S. aureus (MRSA) LAC* strain, a member of the USA300 lineage and a strong producer of SpA, featured strong adhesion signatures similar to those of the Newman strain (Fig. 3C; Fig. S1B). Adhesion forces were strongly reduced in LAC* Δ*spa* mutant cells deficient in SpA (Fig. 3D), implying again they involve SpA proteins. Rupture lengths were larger than with Newman cells (∼300 nm and sometimes even longer), implying that not only SpA but also vWF was elongated upon pulling. Thus, the strong vWF-binding forces measured in the laboratory strain also apply to the clinically relevant strain.

### Mechanical strength of the SpA-vWF complex.

To dissect the mechanical strength of the SpA-vWF complex, Newman cells were probed using single-molecule AFM with vWF-modified tips (Fig. 4A; 3 different cells [for more cells, see Fig. S2 in the supplemental material]). About 15% of the force curves featured adhesion events with a small number of weak adhesion peaks of 107 ± 56 pN and a large fraction of strong peaks of 2,313 ± 217 pN (*n *= 458 adhesive curves from 3 cells). Most adhesion events were abolished with Newman Δ*spa* cells (Fig. 4B), supporting the specificity of the measurements. Mostly single bonds were probed because strong forces showed a narrow distribution and featured single rupture peaks, which is not expected if the number of bonds varied from one pull to another. Rupture lengths featured a more complex pattern than with whole cells (Fig. 3), with values ranging from ∼100 to 400 nm. The extent to which SpA is unfolded upon pulling is not known. However, as the length of the fully unfolded SpA is ∼180 nm (assuming the adhesin is made of ∼500 residues [19]), our values show that vWF contributes to protein extensions. We therefore suggest that the ∼100- to 200-nm and ∼200- to 400-nm ruptures may result from a change in the structure of vWF, from a globular state to an extended chain conformation. Single-molecule imaging suggested that the adhesins form clusters on the cell surface (Fig. 4A to C, insets), a phenomenon that could enhance bacterial adhesion through multivalency.

10.1128/mBio.00555-19.2 FIG S2 Strength of single SpA-vWF bonds. (A) Maximum adhesion force histograms (left) with force maps (insets [image size = 500 nm]) and rupture length histograms (right) obtained by recording force-distance curves in PBS between 3 additional Newman WT bacteria and vWF-modified AFM tips. Download FIG S2, DOCX file, 0.9 MB. Copyright © 2019 Viela et al. 2019 Viela et al. This content is distributed under the terms of the Creative Commons Attribution 4.0 International license.

### Mechanoregulation of the SpA-vWF bond.

**FIG 5:**
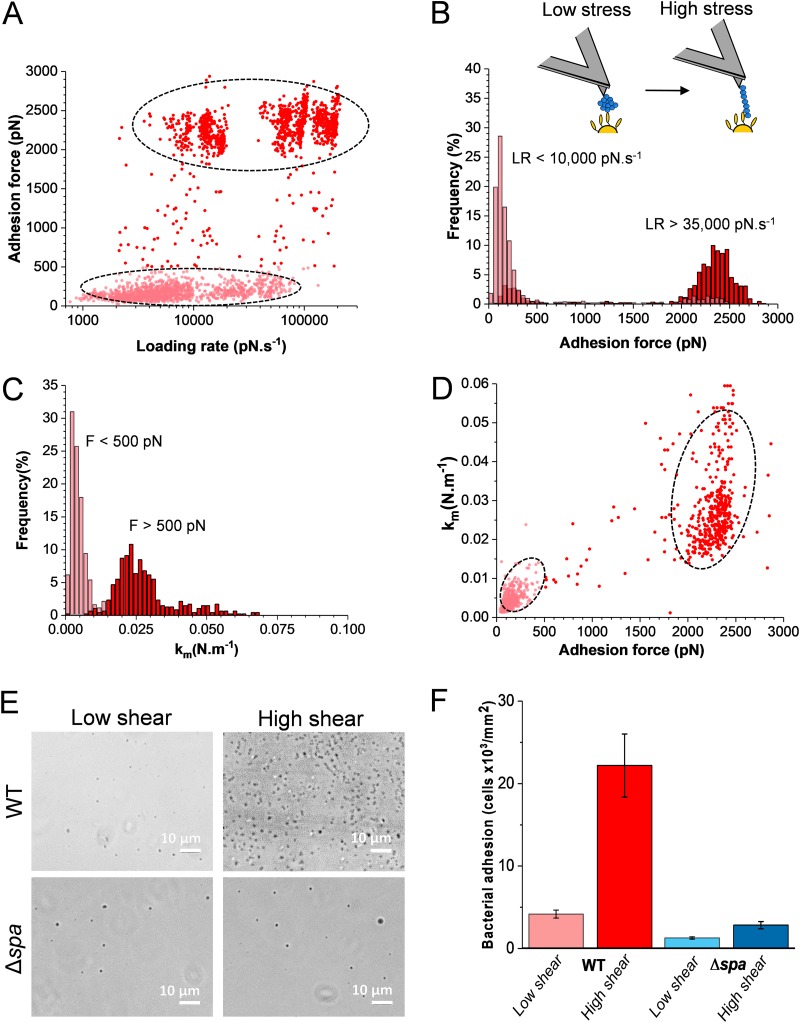
Mechanical activation of vWF binding to SpA. (A) Strength of single SpA-vWF bonds measured at increasing loading rates (LRs) on Newman WT bacteria (2,468 adhesive events from 6 cells). All adhesion peaks were analyzed to take into account all possible interactions. (B) Analysis of the data in panel A showing that strong bonds are favored at high LR. Discrete ranges of LRs were binned, and the forces were plotted as histograms (see Fig. S3 in the supplemental material). (C) Stiffness of the SpA-vWF complex (*k_m_*) at low (<500 pN) and high (>500 pN) tensile forces. A cutoff of 500 pN was chosen as essentially all low adhesion forces at low stress were smaller than this value (see panel B). (D) Plot of *k_m_* as a function of the adhesion force showing that the high mechanical stability of the bond correlates with a high molecular stiffness. (E and F) SpA-dependent bacterial adhesion to vWF increases with fluid shear stress. Shown are optical microscopy images of Newman WT and Δ*spa* bacteria adhering to vWF-coated surfaces in a microparallel flow chamber under low and high shear stresses. Shown in panel F is the quantification of the amounts of adhering bacteria estimated from the experiments described in panel E from a total of 6 images from 3 experiments for each condition.

As vWF binding to S. aureus is influenced by shear stress conditions, we hypothesized that the strength of the SpA-vWF bond might depend on mechanical force. Figure 5A shows the SpA-vWF adhesion force (*F*) measured on Newman bacteria with vWF-modified tips while varying the loading rate (*LR*; assessed from force versus time curves [20, 21]) (data pooled from 2,468 adhesive peaks from 6 cells). Two clouds of data points corresponding to low and high forces can be distinguished. Analysis of the forces over discrete ranges of LRs (Fig. 5B) revealed that the probability of forming strong bonds increases with the LR. At low LRs, forces of 146 ± 68 pN were seen, while at high LRs, forces of 2,359 ± 174 pN dominated. Intermediate forces in the range of 500 to 1,500 pN were never observed. This shift in force distribution demonstrates that the SpA-vWF interaction strengthens under mechanical tension.

10.1128/mBio.00555-19.3 FIG S3 Mechanical force increases the strength of the SpA-vWF bond. Adhesion forces were measured at various loading rates (LRs) between Newman bacteria and vWF-tips. Discrete ranges of LRs were binned, and the force distribution was plotted as histograms (data pooled from 2,468 adhesive events on 6 cells). Download FIG S3, DOCX file, 0.4 MB. Copyright © 2019 Viela et al. 2019 Viela et al. This content is distributed under the terms of the Creative Commons Attribution 4.0 International license.

We also estimated the mechanical stiffness (*k_m_*) of the SpA-vWF molecular complex under low and high tension (Fig. 5C), using the slope (*s*) of the linear portion of the raw deflection versus piezo displacement curves and the following equation: *k_m_*= (*k*_c_ × *s*)/(1 − *s*), where *k*_c_ is the spring constant of the AFM cantilever (21). Weak and strong forces featured values of *k_m_* = 4.2 ± 0.2 pN nm^−1^ and *k_m_* = 24 ± 5 pN nm^−1^, respectively, revealing that the strength and stiffness of the bonds were correlated. This was also evident by plotting the stiffness versus adhesion force of the complex for all force curves (Fig. 5D). These observations suggest that mechanical force induces a structural change in the complex, from a folded, weakly binding state to an extended, strongly binding state.

Lastly, we wondered whether the force-dependent binding of SpA could favor bacterial adhesion under shear flow conditions, as observed with catch bonds (22). To substantiate this further, bacterial adhesion was studied under flow at low and moderate shear stress (Fig. 5E and F). Bacterial suspensions were flowed over vWF-coated substrates mounted into a microparallel flow chamber, and adhering bacteria were directly observed by optical microscopy. While WT bacteria poorly adhered at low shear rate (12 s^−1^), adhesion was largely increased at moderate shear rate (120 s^−1^), corresponding to normal venous shear rates (23). Note that there is no clear definition of what are low and high shear rates. In population studies dealing with S. aureus adhesion, shear rates of about 50 to 100 s^−1^ are generally considered low. Therefore, our shear rate of 120 s^−1^ should probably be considered moderate rather than high. So while AFM data cover a wide range of shear stresses that are relevant to *in vivo* situations, our flow data only cover a small range of shear stress. Adhesion of Δ*spa* cells was low and independent of shear conditions. This shows that SpA-dependent *S aureus* adhesion to vWF under fluid flow is increased by shear stress, as reported for the ClfA-mediated adhesion to vWF in flowing blood (4, 5).

## DISCUSSION

How physical stress regulates cellular functions in S. aureus is a largely unsolved question. Several studies have shown that SpA-dependent S. aureus adhesion to vWF is influenced by fluid shear (12, 24, 25), but the molecular interactions involved have not yet been investigated. Using single-molecule experiments, we have identified a novel mechanism of mechanoregulation of S. aureus adhesion in which SpA-vWF bonds are enhanced by mechanical tension. The newly discovered mechanoregulation mechanism reported here might be of medical relevance as it explains how S. aureus is capable of resisting high shear forces of flowing blood during endovascular infections and how the pathogen attaches to platelets under fluid shear. In pharmacology, the SpA-vWF bond might be an important target for the development of novel therapeutics against S. aureus.

SpA mediates bacterial adhesion to immobilized vWF through specific bonds that are much stronger (∼2 nN) than most receptor-ligand interactions studied to date. Compared to its soluble form, immobilized vWF is biologically important as it promotes platelet adhesion at sites of vascular injury (8). Weaker bonds are detected on endothelial cells, reflecting rupture of vWF weakly anchored in the ECM. Notably, vWF binding to SpA is tightly mechanoregulated: the bond is weak at low tensile force, but is dramatically increased at high force. To understand the molecular origin of the force-dependent SpA-vWF adhesion, it is interesting to compare our results with the forces measured so far for other staphylococcal adhesins, especially the structurally and functionally related Staphylococcus epidermidis SdrG and S. aureus ClfA and ClfB. The structural features and molecular biology of these three proteins have been widely investigated (26). They bind to their ligands (e.g., fibrinogen) via the well-known dock, lock, and latch (DLL) mechanism. The binding site of these adhesins is a cleft of 30 Å in length between the N2 and N3 subdomains located in the A region of the protein. Once the ligand peptide is docked and stabilized by hydrophobic interactions and hydrogen bonds, a C-terminus extension of the N3 subdomain folds over the ligand to insert and complement a β-sheet in the N2 subdomain. This DLL mechanism thus greatly stabilizes the conformation of the complex (27). Single-molecule AFM experiments have shown that SdrG, ClfA, and ClfB display an extreme mechanical stability (20, 21, 28), with a binding strength much larger than those of all biomolecular bonds studied so far. Recently, the Gaub group used simulations to unravel the mechanism behind this extreme mechanostability (29, 30). They found that the target peptide is confined in a screwlike manner in the binding pocket of SdrG and that the binding strength of the complex results from numerous hydrogen bonds between the peptide backbone and SdrG, independent of peptide side chains. Rupture of the complex requires all hydrogen bonds to be broken simultaneously. Interestingly, ClfA and ClfB may bind their ligands through a catch bond as their binding strength increases as mechanical force is applied (20, 21).

Our findings are novel and unique in that SpA is structurally and functionally very different from SdrG and ClfA/ClfB (26), as well as from other adhesins investigated so far, and does not involve a DLL binding mechanism. The force-regulated SpA-vWF interaction is likely to involve conformational changes in vWF, and possibly as well in SpA. vWF is a mechanosensitive protein capable of responding to external forces, such as hydrodynamic shear in flowing blood, to tune its biological functions (31). Hydrodynamic forces induce a change in the structure of vWF, from a globular state to an extended chain conformation with exposure of intramolecular globular domains. This stress-induced elongation promotes the adhesion of vWF to platelets and collagen (see, e.g., reference 32). This leads us to suggest that the force-induced extension of vWF may lead to the exposure of cryptic high-affinity binding sites in vWF domains, to which SpA adhesins on the bacterial cell surface strongly bind. The transition from a globular, weak binding state, to a stretched, strong binding state is supported by the long protein extensions that we observed (up to ∼400 nm versus ∼180 nm expected for unfolded SpA), implying that vWF is indeed extended under force, and by the correlation between the strength and stiffness of the SpA-vWF complex. In flowing blood, bacteria are exposed to high shear conditions, corresponding to loading rates that can be larger than 100,000 pN/s (33 [see the supplemental material]). As the SpA-vWF bond is activated at loading rates of 10,000 pN/s, this mechanism will occur *in vivo*. Our model is reminiscent of the binding of the vWF A1 domain to platelet GPIbα, which is activated through a two-step conformational transition (31): under flow, elongation of vWF from a compact to a linear form leads to the exposure of cryptic binding sites, and then force-induced conformational changes convert the A1 domain from a low-affinity to high-affinity state.

While SpA is not engaged in DLL binding, structural changes in the adhesin may also occur during the force-dependent binding to vWF. SpA includes five small three-helix-bundle domains separated by conserved flexible linkers. The three-dimensional (3D) structure of SpA has only been solved for single domains (34, 35). The relative orientation of successive helical bundles when attached to the bacteria cell has, to our knowledge, not been addressed. Structural studies have revealed that SpA exhibits extensive, unusual multiscale conformational heterogeneity. This structural plasticity could enable SpA to bind multiple partners (34, 35). Analysis of a single domain in complex with an Fc fragment of human IgG shows that structural changes occur in SpA when it binds to Fc, including a significant reduction in conformational heterogeneity as well as displacement of an SpA side chain. These studies suggest that concerted backbone/side-chain changes are used for binding multiple partners. So there might be opportunities for force-induced structural changes in the adhesin domains to favor strong binding to vWF. Clearly, clarification of the molecular nature of the interaction of SpA with vWF requires new structural biology data on the SpA-vWF complex.

## MATERIALS AND METHODS

### Bacterial strains and growth conditions.

We used the laboratory strain Newman and the derivative Newman Δ*spa* mutant deficient in protein A (36). We also studied a MRSA isolate called LAC*, an erythromycin-sensitive variant of LAC that is a strong producer of SpA (37), and the derivative LAC* Δ*spa* mutant deficient in protein A (38). All strains were grown in brain heart infusion (BHI) broth with shaking at 200 rpm at 37°C to stationary phase. For AFM experiments, cells were harvested by centrifugation at 2,500 × *g* for 5 min and washed twice with phosphate-buffered saline (PBS).

### Cell culture.

Human umbilical vein endothelial cells (HUVECs) from a single donor (Lonza, Spain) were cultured in T25 flasks (Becton, Dickinson, Germany) and incubated at 37°C in 5% CO_2_ with 100% humidity. Commercial basal medium, supplemented with growth factors and cytokines (EGM BulletKit; Lonza) was used. Cells from passages 3 to 8 were seeded on 35-mm glass-bottom petri dishes 48 h prior to the experiments and were used after reaching confluence. For immunofluorescence experiments, cells were cultured on 10-mm glass coverslips. In order to stimulate vWF expression, cells were treated for 30 min with 5 μM calcium ionophore A23187 (Sigma-Aldrich).

### Immunofluorescence microscopy.

For visualization of vWF in the ECM, cells were fixed with 4% paraformaldehyde in PBS for 10 min and permeabilized at room temperature with 0.1% Triton X-100 (Sigma-Aldrich). To prevent nonspecific antibody binding, endothelial cells were pretreated with 10% bovine serum albumin (Sigma) at room temperature for 60 min and then incubated overnight at 4°C with mouse anti-vWF antibody (MA5-14029; Thermo Fisher Scientific). After being washed with PBS, a secondary goat anti-mouse Alexa Fluor 647 antibody (Abcam) was added for 60 min. Furthermore, all samples were stained with DAPI (4′,6-diamidino-2-phenylindole) to facilitate the identification of the correct focal plane. Finally, coverslips were mounted in mounting medium (Dako, Germany). Fluorescent images were acquired using a Zeiss LSM 710 confocal laser scanning microscope, equipped with a 63× NA1.4 HC PL APO CS2 oil immersion objective. The fluorophores were excited at 561 or 405 nm, respectively. Sequential scanning was applied to avoid concurrent fluorescence signals from two fluorophores.

For the bacterial adhesion assay, confluent HUVECs, being in culture for at least 48 h, were coincubated with S. aureus bacterial cells in stationary phase (50 μl of a diluted cell suspension per 1 ml of medium), pretreated with LIVE/DEAD BacLight bacterial viability kit (Thermo Fisher Scientific) for 15 min at room temperature. After intensive washing of the sample, three-dimensional analysis using a Zeiss LSM 710 confocal laser scanning microscope was performed. The fluorophores were excited at 488 nm.

### Bacterial adherence to vWF-coated microtiter wells.

Microtiter wells were coated overnight at 4°C with 1 μg/well human vWF in 0.1 M sodium carbonate (pH 9.5). The plates were washed with PBS containing 0.5% (vol/vol) Tween 20 (PBST). To block additional protein-binding sites, the wells were treated for 1 h at 22°C with 2% (vol/vol) bovine serum albumin (BSA) in PBS. To test the effect of soluble protein A, the assay was performed in the presence of 2 μg recombinant SpA (Thermo Fisher Scientific). The wells were then incubated for 1 h at 37°C with 1 × 10^8^ cells of the S. aureus Newman strain or its Δ*spa* mutant. After being washed with PBS, adhering cells were fixed with 2.5% formaldehyde for 30 min and stained with 1% crystal violet for 1 min. Following further washing, 100 ml of 10% acetic acid was added, and absorbance at 595 nm was recorded using an enzyme-linked immunosorbent assay (ELISA) plate reader (Bio-Rad).

### Quantification of vWF release by ELISA.

HUVEC monolayers were incubated with the calcium ionophore A23187 for 10 min. The wells were washed three times with PBS and then fixed with 4% paraformaldehyde for 10 min at 4°C. Wells were rinsed with PBS, blocked with 2% BSA in PBS for 1 h at 22°C, and incubated with 1:1,000-diluted rabbit anti-vWF polyclonal antibodies for 1 h at 22°C. The plates were washed and then incubated for 1 h with horseradish peroxidase (HRP)-conjugated goat anti-rabbit IgG diluted 1:1,000. After further washing, *o*-phenylenediamine dihydrochloride was added, and the absorbance at 490 nm was determined using an ELISA plate reader.

### Endothelial cell adhesion assays.

Confluent monolayers of HUVECs were washed three times with PBS to remove antibiotics and treated or not for 10 min with 1 μM calcium ionophore A23187. Cell monolayers were then washed 3 times with PBS and incubated with 10^7^ bacterial cells for 1 h at 37°C. To determine the total number of associated CFU (adherent and internalized), each well was washed 3 times with PBS and cells were lysed by the addition of 0.1% Triton X-100 solution. Bacterial CFU were counted the day after by serial dilution of endothelial cell lysates and plating onto tryptic soy agar (TSA) plates.

### Bacterial adhesion to vWF-coated surfaces.

Adhesion of Newman and Newman Δ*spa* bacteria was assessed on vWF-functionalized surfaces under static and dynamic conditions. For static experiments, bacterial suspensions in PBS were incubated with vWF surfaces for 2 h at 37°C, gently rinsed with PBS, and imaged using an optical microscope Zeiss Axio Observer Z1 and a Hamamatsu camera C10600. For flow experiments, bacterial suspensions were flowed over vWF surfaces during 3 min using a fluidic chamber (39), using a peristaltic pump (Miniplus, Gilson). Two different flow rates were tested (2 and 20 ml min^−1^), corresponding to shear rates of 12 and 120 s^−1^, respectively. Loosely attached bacteria were removed by flowing PBS during 3 min using the corresponding flow rate. Adhering bacteria were imaged using an inverted microscope (Leica DM16000) and counted using the Image J image analysis software (NIH Image).

### Functionalization of substrates and cantilevers with vWF.

Gold-coated glass coverslips and cantilevers (OMCL-TR4, Olympus Ltd., Tokyo, Japan; nominal spring constant, ∼0.02 N m^−1^) were immersed overnight in an ethanol solution containing 1 mM 10% 16-mercaptododecahexanoic acid–90% 1-mercapto-1-undecanol (Sigma), rinsed with ethanol, and dried with N_2_. Substrates and cantilevers were then immersed for 30 min into a solution containing 10 mg ml^−1^ *N*-hydroxysuccinimide (NHS) and 25 mg ml^−1^ 1-ethyl-3-(3-dimethylaminopropyl)-carbodiimide (EDC [Sigma]), rinsed with Ultrapure water (ELGA LabWater), incubated with 0.1 mg ml^−1^ vWF (Merck) for 1 h, rinsed further with PBS buffer, and then immediately used without dewetting.

### Single-cell force spectroscopy on endothelial cells.

Colloidal probes were obtained by attaching single silica microspheres (6.1-μm diameter; Bangs Laboratories) with a thin layer of UV-curable glue (NOA 63, Norland Edmund Optics) to triangular shaped tip-less cantilevers (NP-O10; Bruker) using a Nanowizard III atomic force microscope (JPK Instrument, Berlin, Germany). Cantilevers were then immersed for 1 h in Tris-buffered saline (TBS; Tris, 50 mM; NaCl, 150 mM; pH 8.5) containing 4 mg ml^−1^ dopamine hydrochloride (Sigma-Aldrich), rinsed in TBS, and used directly for cell probe preparation. The nominal spring constant of the colloidal probe was determined by the thermal noise method. Then, 50 μl of a diluted cell suspension was deposited into the petri dish containing HUVECs at a distinct location within the petri dish; 1 ml of HEPES buffer (HEPES, 10 mM; glucose, 5 mM; MgCl_2_, 1 mM; KCl, 5 mM; NaCl, 140 mM; pH 7.4) was added to the system. The colloidal probe was put in contact with a single bacterial cell and retracted to attach it on the silica microsphere; proper attachment of the cell on the colloidal probe was checked using optical microscopy. Cell probes were used to measure interaction forces on HUVECs at room temperature, and adhesion maps were obtained by recording 8-by-8 or 16-by-16 force-distance curves on 1- by 1-μm lamellopodia areas of the cells, using an applied force of 250 pN, a constant approach-retraction speed of 1,000 nm s^−1^ (for the Nanowizard III AFM system), and a contact time of 250 ms. Data were analyzed using the Data Processing software from JPK Instruments (Berlin, Germany). Adhesion force and rupture distance histograms were obtained by calculating the maximum adhesion force and the rupture distance of the last peak for each curve.

### Single-cell force spectroscopy on vWF surfaces.

Colloidal probes were prepared as described above, using a Nanoscope VIII multimode atomic force microscope (Bruker Corporation, Santa Barbara, CA). The nominal spring constants of the colloidal probe cantilevers were determined by the thermal noise method. For cell probe preparation, 50 μl of a suspension of ca. 1 × 10^6^ cells was transferred into a glass petri dish containing vWF-coated substrates in PBS. The colloidal probe was brought into contact with a bacterium. Single bacteria were attached on the center of the colloidal probes using a Nanowizard III atomic force microscope (JPK Instruments). The cell probe was then positioned over the vWF substrate without dewetting. Cell probes were used to measure interaction forces on vWF surfaces at room temperature by recording multiple force curves (16 by 16) on different spots using a contact time of 250 ms, a maximum applied force of 250 pN, and approach and retraction speeds of 1,000 nm s^−1^. Adhesion force and rupture distance histograms were obtained by calculating the maximum adhesion force and the rupture distance of the last peak for each curve.

### Single-molecule force spectroscopy on living bacteria.

Bacteria were immobilized by mechanical trapping in porous polycarbonate membranes (Millipore, Billerica, MA) with a pore size similar to the cell size (for details, see reference 40). After ﬁltration of a cell suspension was performed, the ﬁlter was gently rinsed with PBS, carefully cut into sections (1 cm by 1 cm), and attached to a steel sample puck using a small piece of double-sided adhesive tape, and the mounted sample was transferred into the AFM liquid cell while avoiding dewetting. Analyses were performed on live bacteria at room temperature (20°C) in PBS using a Nanoscope VIII multimode atomic force microscope (Bruker Corporation, Santa Barbara, CA) and gold-coated cantilevers (Olympus OTR4 with a *k* value of ∼0.02 N m^−1^). The spring constants of the cantilevers were measured using the thermal noise method. Arrays of 32-by-32 force curves were recorded on 500- by 500-nm areas using a contact time of 250 ms, a maximum applied force of 250 pN, and approach and retraction speeds of 1,000 nm s^−1^. For loading rate experiments, arrays of 32-by-32 force curves were recorded on 500- by 500-nm areas at increasing retraction speeds: 0.5, 1, 5 and 10 μm s^−1^. Adhesion and rupture length histograms were generated by considering, for every force curve, the maximum adhesion force and the rupture distance of the last peak. For loading rate experiments, all adhesion peaks were analyzed to take into account all possible interactions. Data were analyzed using the Nanoscope Analysis software from Bruker (Santa Barbara, CA, USA).
