# Supplementary material for: Binding of Staphylococcus aureus Protein A to von Willebrand Factor Is Regulated by Mechanical Force
Source: mBio. 2019 Apr 30;10(2):e00555-19. doi: 10.1128/mBio.00555-19 (PMC6495375; doi:10.1128/mBio.00555-19)
Supplement: TABLE S1 [file mBio.00555-19-st001.docx]

**Table S1. Adhesion forces between *S. aureus* and vWF in the ECM of endothelial cells.** Probability of adhesion (P_adh_ = % of curves with adhesion forces), maximum adhesion force (F_adh_) and rupture length (L_rupt_) measured between single Newman WT or Newman Δ*spa* bacteria and HUVEC cells treated or not with calcium ionophore.

|  | | | **w/ ionophore** | | | | | | |  | **w/o ionophore** | | | | | | |
| --- | --- | --- | --- | --- | --- | --- | --- | --- | --- | --- | --- | --- | --- | --- | --- | --- | --- |
|  | | | **P_adh_ (%)** | **F_adh_ (pN)** | | | **L_rupt_ (nm)** | | |  | **P_adh_ (%)** | **F_adh_(pN)** | | | **L _rupt_ (nm)** | | |
| **WT** | cell1 | * | 19 | 133 | ± | 75 | 2294 | ± | 386 |  | 8 | 40 | ± | 19 | 1627 | ± | 1129 |
|  | cell2 | * | 18 | 113 | ± | 64 | 1391 | ± | 1085 |  | 3 | 26 | ± | 18 | 1235 | ± | 568 |
|  | cell3 | * | 50 | 67 | ± | 35 | 846 | ± | 430 |  | 10 | 53 | ± | 50 | 1315 | ± | 633 |
|  | cell4 | * | 34 | 71 | ± | 39 | 946 | ± | 595 |  | 14 | 39 | ± | 17 | 833 | ± | 699 |
|  | cell5 | * | 18 | 89 | ± | 43 | 871 | ± | 823 |  | 5 | 54 | ± | 22 | 1458 | ± | 1074 |
|  | cell6 | * | 10 | 86 | ± | 42 | 2504 | ± | 2073 |  | 4 | 22 | ± | 10 | 1790 | ± | 1239 |
|  | cell7 | * | 10 | 110 | ± | 55 | 3873 | ± | 585 |  | 4 | 37 | ± | 17 | 824 | ± | 382 |
|  | cell8 | * | 64 | 97 | ± | 58 | 971 | ± | 472 |  | 8 | 22 | ± | 10 | 1200 | ± | 392 |
|  | cell9 | * | 15 | 260 | ± | 447 | 1840 | ± | 464 |  | 8 | 29 | ± | 12 | 985 | ± | 473 |
|  | cell10 | * | 30 | 109 | ± | 46 | 1257 | ± | 1080 |  |  |  |  |  |  |  |  |
|  | **mean** | ***** | **27 ± 18** | **114** | **±** | **55** | **1679** | **±** | **974** |  | **7 ± 4** | **36** | **±** | **12** | **1252** | **±** | **337** |
| **Δ*spa*** | cell1 | * | 3 | 42 | ± | 10 | 1243 | ± | 702 |  | 2 | 33 | ± | 13 | 1480 | ± | 571 |
|  | cell2 | * | 3 | 22 | ± | 11 | 1020 | ± | 369 |  | 4 | 39 | ± | 15 | 1433 | ± | 556 |
|  | cell3 | * | 2 | 25 | ± | 10 | 1286 | ± | 471 |  | 7 | 40 | ± | 12 | 1573 | ± | 908 |
|  | cell4 | * | 2 | 27 | ± | 14 | 633 | ± | 350 |  | 6 | 33 | ± | 21 | 1182 | ± | 945 |
|  | **mean** |  | **3 ± 0.6** | **29** | **±** | **9** | **1046** | **±** | **299** |  | **5 ± 2** | **36** | **±** | **4** | **1417** | **±** | **167** |
