# Supplementary material for: Binding of Staphylococcus aureus Protein A to von Willebrand Factor Is Regulated by Mechanical Force
Source: mBio. 2019 Apr 30;10(2):e00555-19. doi: 10.1128/mBio.00555-19 (PMC6495375; doi:10.1128/mBio.00555-19)
Supplement: FIG S1 [file mBio.00555-19-sf001.docx]

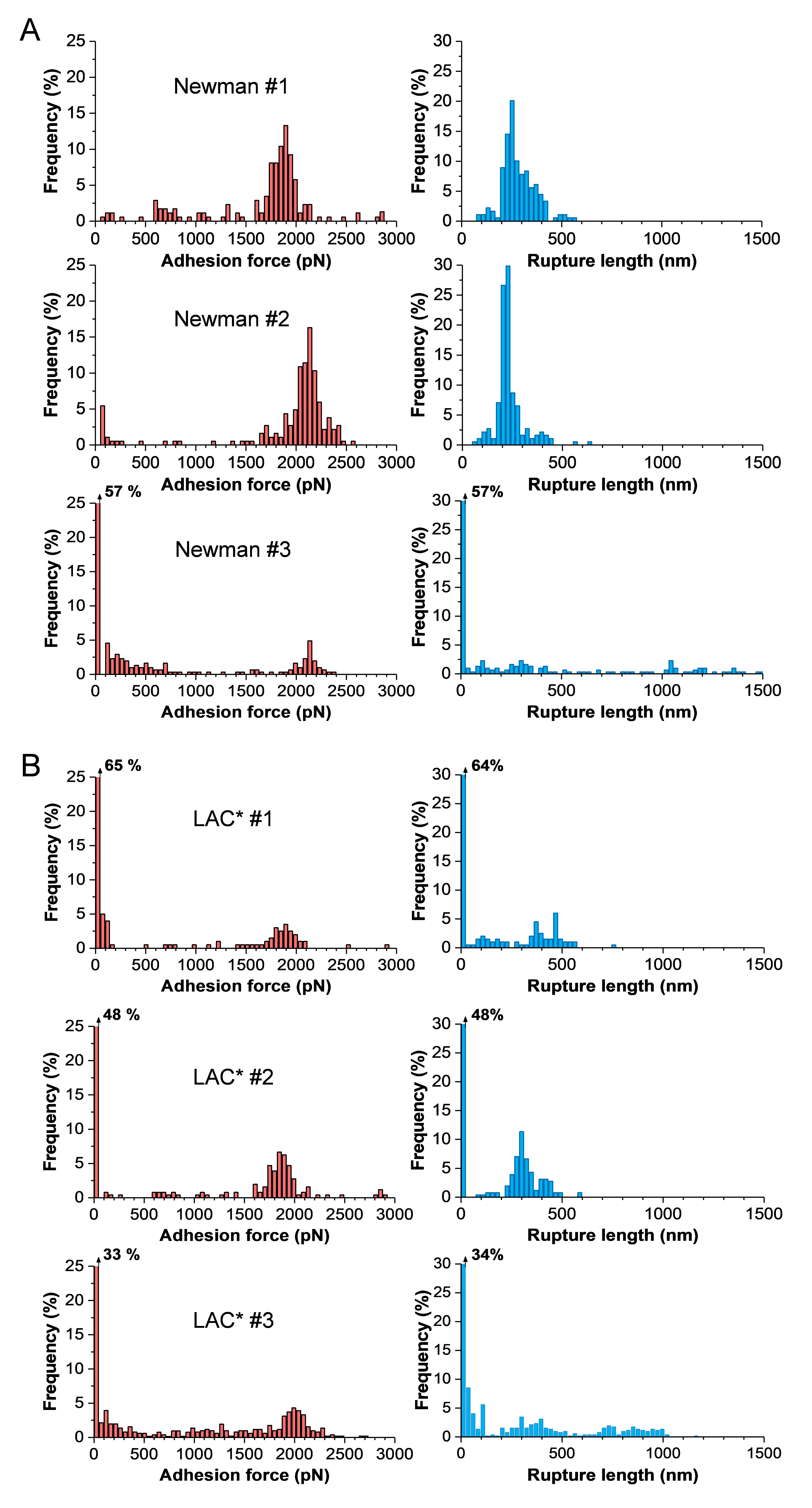


**Figure S1. Adhesion forces between *S. aureus* and immobilized vWF.** (*A*, *B*) Maximum adhesion force (left) and rupture length (right) histograms obtained by recording force-distance curves in PBS between three additional Newman WT (*A*) or LAC* (*B*) bacteria and vWF-coated substrates.
