# Supplementary material for: Binding of Staphylococcus aureus Protein A to von Willebrand Factor Is Regulated by Mechanical Force
Source: mBio. 2019 Apr 30;10(2):e00555-19. doi: 10.1128/mBio.00555-19 (PMC6495375; doi:10.1128/mBio.00555-19)

**Figure S2. Strength of single SpA-vWF bonds.** (*A*) Maximum adhesion force histograms (left) with force maps (insets; image size = 500 nm), and rupture length histograms (right) obtained by recording force-distance curves in PBS between 3 additional Newman WT bacteria and vWF-modified AFM tips.


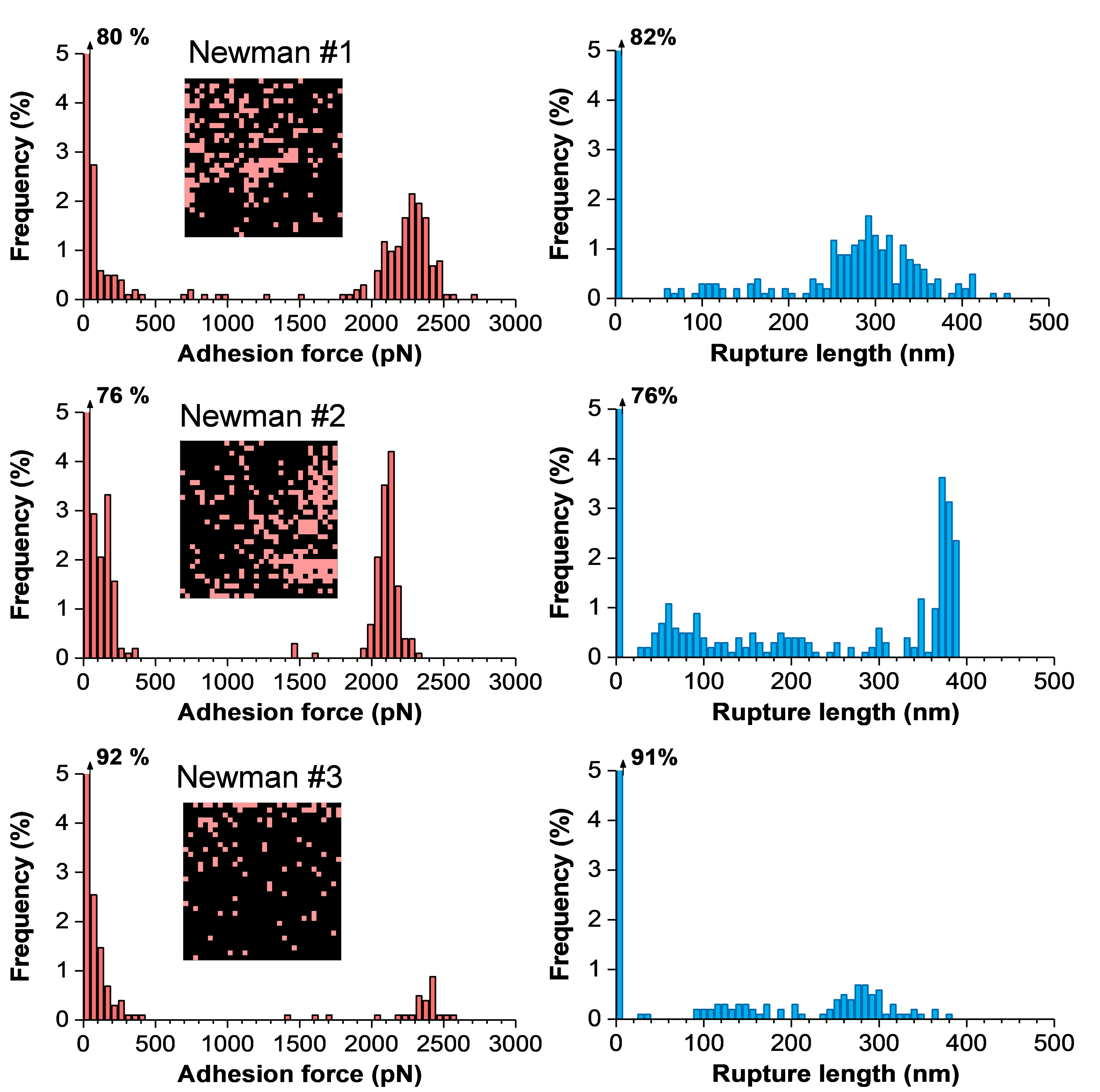

Supplement: FIG S2 [file mBio.00555-19-sf002.docx]
