# Supplementary material for: Binding of Staphylococcus aureus Protein A to von Willebrand Factor Is Regulated by Mechanical Force
Source: mBio. 2019 Apr 30;10(2):e00555-19. doi: 10.1128/mBio.00555-19 (PMC6495375; doi:10.1128/mBio.00555-19)
Supplement: FIG S3 [file mBio.00555-19-sf003.docx]

**
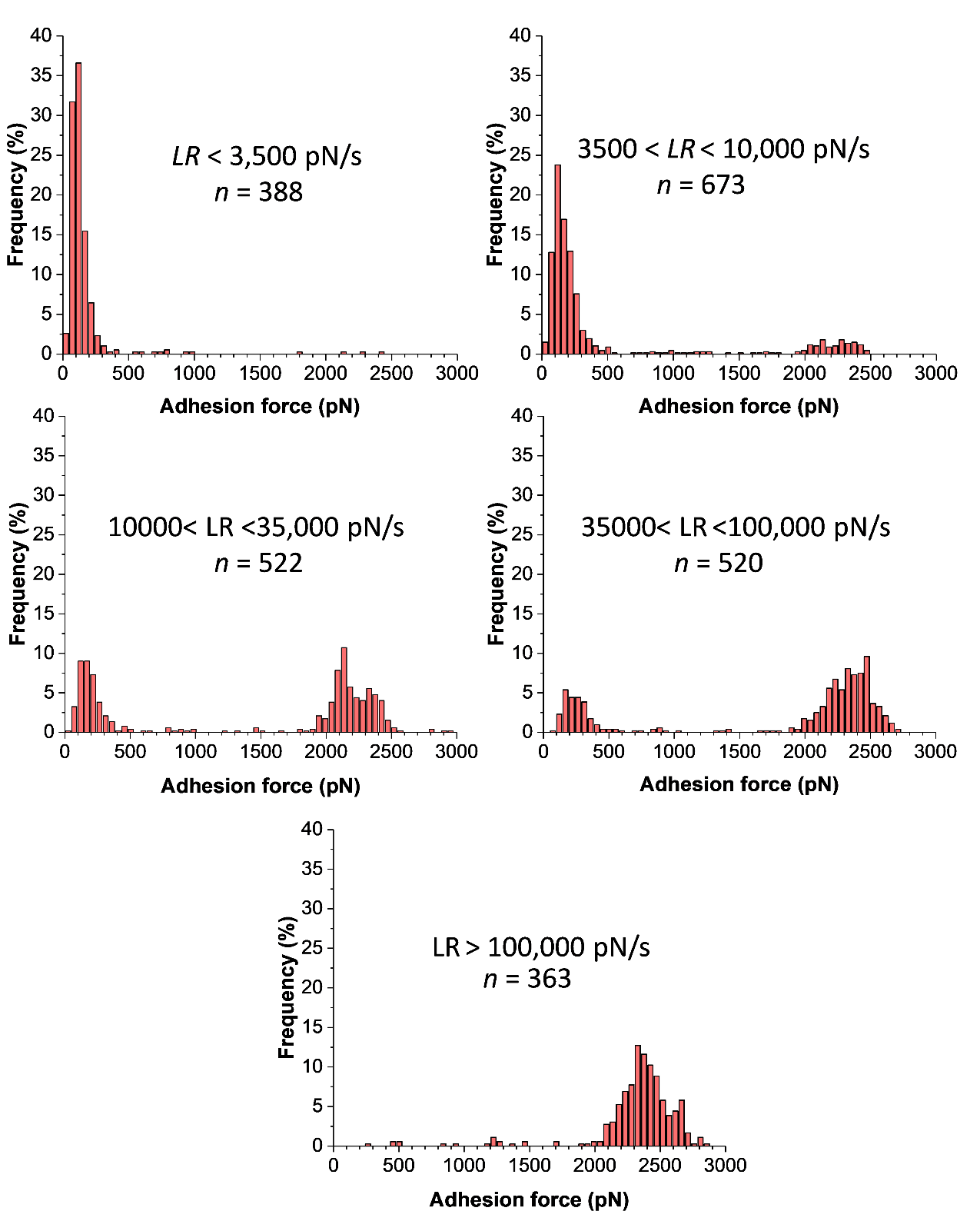
**

**Figure S3. Mechanical force increases the strength of the SpA-vWF bond.** Adhesion forces were measured at various loading rates (*LRs*) between Newman bacteria and vWF-tips. Discrete ranges of *LRs* were binned and the force distribution plotted as histograms (data pooled from 2,468 adhesive events on 6 cells).
